# Risk factors of breast cancer recurrence in pathologic complete response achieved by patients following neoadjuvant chemotherapy: a single-center retrospective study

**DOI:** 10.3389/fonc.2023.1230310

**Published:** 2023-10-02

**Authors:** Joon Young Choi, Doyoun Woen, Sung Yoon Jang, Hyunjun Lee, Dong Seung Shin, Youngji Kwak, Hyunwoo Lee, Byung Joo Chae, Jonghan Yu, Jeong Eon Lee, Seok Won Kim, Seok Jin Nam, Jai Min Ryu

**Affiliations:** ^1^ Division of Breast Surgery, Department of Surgery, Samsung Medical Center, Sungkyunkwan University School of Medicine, Seoul, Republic of Korea; ^2^ Department of Surgery, Samsung Medical Center, Sungkyunkwan University School of Medicine, Seoul, Republic of Korea; ^3^ Department of Pathology and Translational Genomics, Samsung Medical Center, Sungkyunkwan University School of Medicine, Seoul, Republic of Korea

**Keywords:** breast neoplasm, neoadjuvant chemotherapy (NAC), pathologic complete response (pCR), risk factor, recurrence

## Abstract

**Objective:**

Pathologic complete response (pCR) of breast cancer after neoadjuvant chemotherapy (NAC) is highly related to molecular subtypes. Patients who achieved tumor pCR after NAC have a better prognosis. However, despite of better prognosis, pCR patients have a potential for recurrence. There is little evidence of risk factors of recurrence in patients with pCR. We aim to analyze factors associated with tumor recurrence in patients who achieved pCR.

**Methods:**

This study retrospectively reviewed the data of patients diagnosed with breast cancer who achieved pCR after receiving NAC between January 2009 and December 2018 in Samsung Medical Center. pCR was defined as no residual invasive cancer in the breast and axillary nodes even if there is residual ductal carcinoma *in situ* (ypT0 or ypTis with ypN0). Breast cancers are classified into 4 subtypes based on hormone receptors (HR) and human epithelial growth factor receptor 2 (HER2) status. Patients who had bilateral breast cancer, ipsilateral supraclavicular or internal mammary lymph node metastasis, inflammatory breast cancer, distant metastasis, unknown subtype, and histologically unique case were excluded from the study.

**Results:**

In total 483 patients were included in this study except for patients who corresponded to the exclusion criteria. The median follow-up duration was 59.0 months (range, 0.5-153.3 months). Breast cancer recurred in 4.1% of patients (20 of 483). There was a significant difference in clinical T (P = 0.004) and clinical N (P = 0.034) stage in the Kaplan-Meier curve for disease-free survival. Molecular subtypes (P = 0.573), Ki67 (P = 1.000), and breast surgery type (P = 0.574) were not associated with tumor recurrence in patients who achieved pCR after NAC. In the clinical T stage and clinical N stage, there was a significant difference between recurrence and no-recurrence groups (clinical T stage; P = 0.045, clinical N stage; P = 0.002). Univariable Cox regression revealed statistical significance in the clinical T stage (P = 0.049) and clinical N stage (P = 0.010), while multivariable Cox regression demonstrated non-significance in the clinical T stage (P = 0.320) and clinical N stage (P = 0.073).

**Conclusion:**

Results in this study showed that clinical T, clinical N stage, and molecular subtypes were not statistically significant predictors of recurrence in patients who achieved pCR after NAC. In spite of that, pCR after NAC may be more important than clinical staging and molecular subtype in early breast cancer. In addition, escalated treatments for patients with HER2 + or triple-negative tumors would be considered with a strict patient selection strategy to prevent over-treatment as well as achieve pCR.

## Introduction

Neoadjuvant chemotherapy (NAC) as a treatment for advanced breast cancer is generally practiced to increase the rate of breast-conserving surgery and minimize axillary surgery (1, 2). Also, NAC is advantageous in the assessment of the chemosensitivity of cancer (3). According to former studies and randomized trials, the pathologic complete response (pCR) achieving rate after NAC ranges from 17% to 66%; the percentage varies mostly because of molecular subtypes and the different NAC regimens. Trastuzumab or pertuzumab for human epithelial growth factor receptor 2 (HER2) + tumor has demonstrated significantly improved pCR rate (4–6). Importantly, patients who achieved tumor pCR after NAC have a higher opportunity of disease-free survival (DFS) and overall survival (OS) (7, 8).

However, despite a higher opportunity to achieve pCR in patients who received NAC, a proportion of pCR achieved breast cancer still recurs (13-25%) (9, 10). Hence, it is necessitated to escalate the treatments after NAC for a portion of pCR-achieved patients with associated risk factors of recurrence. According to recent randomized control trials (RCTs), escalated treatments such as capecitabine, atezolizumab, sacituzumab, and pembrolizumab for non-pCR triple negative breast cancer (TNBC) and trastuzumab emtansine (T-DM1) for non-pCR HER2 + cancer after NAC led to successful improvement in the oncologic outcomes (11–15). Also, these escalated treatments are believed to aid pCR-achieved patients in preventing cancer recurrence. However, these treatments are not the standard treatments for pCR-achieved patients due to a lack of sufficient evidence.

Therefore, it is hypothesized that the ascertainment of factors related to the recurrence of pCR achieving breast cancer after NAC would lead to recognition of the high-risk patients of breast cancer recurrence and aid in the escalation of treatments. We aimed to investigate the factors associated with breast cancer recurrence in pCR-achieving patients after NAC.

## Methods

### Patients’ selection

We retrospectively reviewed the data of patients diagnosed with breast cancer who underwent a breast surgery at Samsung Medical Center between January 2009 and December 2018. Patients who achieved pCR after receiving preoperative NAC were included. Patients who were diagnosed with bilateral breast cancer and inflammatory breast cancer were excluded. In addition, patients who had ipsilateral supraclavicular or internal mammary lymph node metastasis, distant metastasis, unknown subtype, and histologically unique case like neuroendocrine differentiation were excluded.

### Definition

The factors like age at diagnosis, type of surgery, clinical T stage and clinical N stage of tumors before receiving NAC, the molecular subtype of tumors, hormone receptors (HR) status (estrogen receptor (ER) status or progesterone receptor (PR) status), HER2 status and Ki67 expression, and axillary node metastasis were included in the study. The positivity of ER, PR, and HER2 was determined according to the American Society of Clinical Oncology/College of American Pathologists (ASCO/CAP) guidelines (16). ER status and PR status were assessed by immunohistochemistry (IHC) and categorized as positive if there were at least 1% of stained cancer cells. HER2 was considered positive if there was evidence of protein overexpression (immunohistochemistry staining 3+) or gene amplification (fluorescent *in situ* hybridization with a HER2/CEP17 ratio ≥ 2 or average HER2 copy number ≥ 6 signals/cell) (17). HER2-low is defined as HER2 immunohistochemistry (IHC) score of 1+ or 2+ and not amplification in *in situ* hybridization (ISH) according to European society for medical oncology (ESMO) expert consensus statements (ECS) (18). A low level of Ki67 expression was indicated as a percentage of cells with positive nuclei staining < 20% and a high level of Ki67 expression as ≥ 20% (19). Axillary node positivity was determined by cytological metastasis in axillary fine needle aspiration biopsy. Breast cancers were classified into 4 subtypes based on the HR and HER2 status as follows: HR +/HER2 –, HR +/HER2 +, HR –/HER2 +, and triple-negative subtype (both HR – and HER2 –). pCR was defined as no residual invasive cancer in the breast and axillary nodes even if there is residual ductal carcinoma *in situ* (ypT0/is/ypN0). A locoregional recurrence (LRR) was defined as a local recurrence and a regional recurrence. A local recurrence was defined as a recurrence of ipsilateral breast, chest wall, and skin. A regional recurrence was defined as a recurrence of ipsilateral axillary, internal mammary, infraclavicular, and supraclavicular lymph nodes. A distant metastasis included only contralateral axillary, internal mammary, infraclavicular, and supraclavicular lymph nodes without contralateral breast cancer. Contralateral breast cancer was not included in the recurrence.

### Statistical analyses

Patients’ characteristics were compared using the Chi-square test and Fisher’s exact test for categorical variables. Risk factor analysis was conducted using multivariable analysis with logistic regression. Statistical significance was established at P < 0.050. All statistical analyses were performed using the Statistical Analysis System (SAS) version 9.4 (SAS Institute Inc., Cary, NC, USA) and International Business Machines Corporation Statistical Package for the Social Sciences (IBM SPSS) Statistics Version 27.

### IRB number

This study adhered to the ethical tenets of the Declaration of Helsinki and was approved by the Institutional Review Board (IRB) of SMC (IRB number: 2022-08-139). The need for informed consent was waived because of the retrospective nature of the study.

## Results

### Patients’ and tumor characteristics

**Figure 1:**
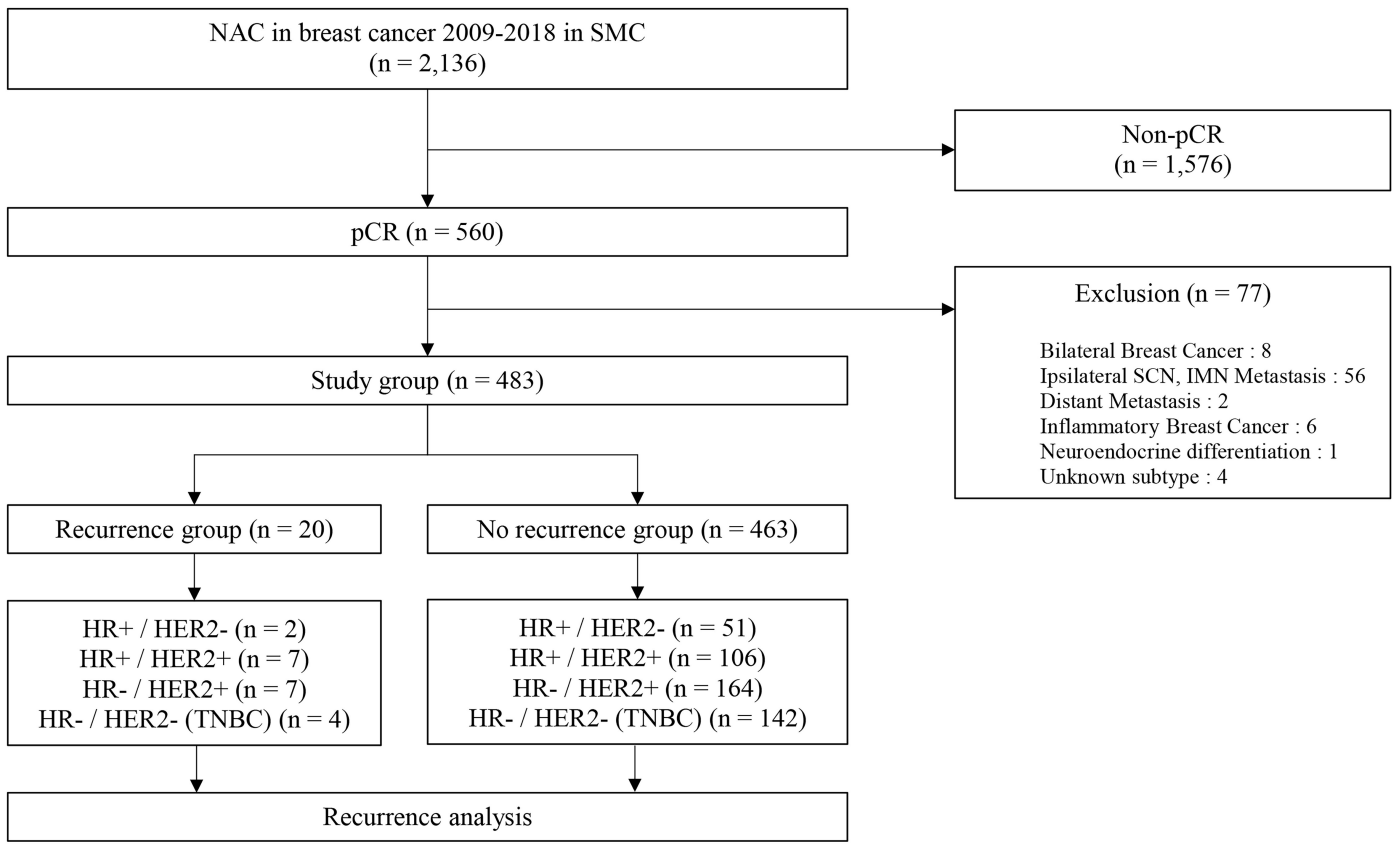
Schematic diagram for the inclusion of participants. NAC, neoadjuvant chemotherapy; SMC, Samsung medical center; pCR, pathologic complete response; SCN, supraclavicular node; IMN, internal mammary node.

Among the 2,316 included patients, a total of 560 patients who were diagnosed with breast cancer achieved pCR after receiving NAC between January 2009 and December 2018 at Samsung Medical Center. In total, 483 patients were included in this study except for patients who corresponded to the exclusion criteria. Of 483 patients in the study group, 20 patients had a recurrence of breast cancer (recurrence group) and 463 patients had no recurrence (no recurrence group) (**Figure 1**). The median follow-up duration was 59.0 months (range, 0.5-153.3 months). Most of the patients had clinical T2 stage in recurrence (50%) and no recurrence (68.5%) groups. In the recurrence group, most of the patients had clinical N2 stage (40%) and a maximum number of patients in the no recurrence group had clinical N0 stage (35.9%). There was a significant difference between the recurrence and no recurrence groups at the clinical T stage (P = 0.045 and clinical N stage (P = 0.002). Typically, 10% of the recurrence group and 11% of the no-recurrence group had types of HR+/HER2 – breast cancer. Whereas, 70% of the recurrence group and 58.3% of the other group had HER2 positivity. The proportion of triple-negative breast cancer (TNBC) was 20% in the recurrence group and 30.7% in the no-recurrence group. However, there was no statistically significant difference in the correlation between molecular subtypes and recurrence risk in patients who achieved pCR after NAC (P = 0.573). Ki67 (P = 1.000) and breast surgery type (P = 0.574) were also not associated with tumor recurrence (**Table 1**). The additional clinicopathologic characteristics of patients with HER2-low was disclosed with a supplement. Total patients in the HER2-low group were 73 and the mean f/u duration was 58.1 months (**Table S1**).

**Table 1:** Clinicopathologic characteristics of patients.

Characteristics	Recurrence No. (%) N = 20 (4.1)	No Recurrence No. (%) N = 463 (95.9)	%	P value
**Mean F/U duration (month)**	59.0 (0.5 - 153.3)			
**Age at diagnosis (years)**				0.162
≤ 35	2 (10.0)	58 (12.5)	12.4	
36 - 50	6 (30.0)	226 (48.8)	48.0	
> 50	12 (60.0)	179 (38.7)	39.5	
**Clinical T stage**				0.045
cT0 or Tis or T1	2 (10.0)	42 (9.1)	9.1	
cT2	10 (50.0)	317 (68.5)	67.7	
cT3	6 (30.0)	97 (21.0)	21.3	
cT4	2 (10.0)	7 (1.5)	1.9	
**Clinical N stage**				0.002
cN0	3 (15.0)	166 (35.9)	35.0	
cN1	5 (25.0)	140 (30.2)	30.0	
cN2	8 (40.0)	141 (30.5)	30.8	
cN3	4 (20.0)	16 (3.5)	4.1	
**FNA of metastatic lymph node**				0.450
negative by proven Bx	4 (20.0)	106 (22.9)	22.8	
positive by proven Bx	13 (65.0)	235 (50.8)	51.3	
Did not Bx	3 (15.0)	122 (26.3)	25.9	
**Molecular subtype at diagnosis**				0.573
HR+/HER2-	2 (10.0)	51 (11.0)	11.0	
HR+/HER2+	7 (35.0)	106 (22.9)	23.4	
HR-/HER2+	7 (35.0)	164 (35.4)	35.4	
HR-/HER2- (TNBC)	4 (20.0)	142 (30.7)	30.2	
**Ki67 at diagnosis**				1.000
< 20%	2 (10.0)	51 (11.0)	11.0	
≥ 20%	18 (90.0)	407 (87.9)	88.0	
Unknown	0 (0.0)	5 (1.1)	1.0	
**Breast surgery**				0.574
Mastectomy	5 (25.0)	93 (20.1)	20.3	
BCS	15 (75.0)	370 (79.9)	79.7	
**Axillary surgery**				0.189
SLNB only	12 (60.0)	346 (74.7)	74.1	
ALND	8 (40.0)	117 (25.3)	25.9	
**Adjuvant RT**				0.665
Yes	18 (90.0)	427 (92.2)	92.1	
No	2 (10.0)	36 (7.8)	7.9	
**NAC regimen**				0.020
AC	0 (0.0)	13 (2.8)	2.7	
T	0 (0.0)	4 (0.9)	0.8	
AC+T	9 (45.0)	240 (51.8)	51.6	
ACTH	5 (25.0)	19 (4.1)	5.0	
TCHP	6 (30.0)	139 (30.0)	30.0	
Others	0 (0.0)	48 (10.4)	9.9	

F/U, follow-up; FNA, fine needle aspiration; Bx, biopsy; HR, hormone receptors; HER2, human epithelial growth factor receptor 2; TNBC, triple-negative breast cancer; BCS, breast-conserving surgery; SLNB, sentinel lymph node biopsy; ALND, axillary lymph node dissection; RT, radiotherapy; NAC, neoadjuvant chemotherapy; AC, Adriamycin Cyclophosphamide; T, Taxane; AC+T, Adriamycin Cyclophosphamide + Taxane; ACTH, Adriamycin Cyclophosphamide Taxane Herceptin(trastuzumab); TCHP, Taxane Carboplatin Herceptin(trastuzumab) Perjeta(pertuzumab).

### Clinicopathologic factors associated with RFS

Out of 483 patients who achieved pCR in the study group, 20 patients eventually developed a recurrence of cancer. In a univariable Cox regression analysis, the clinical T stage (P = 0.049) and clinical N stage (P = 0.010) were identified to be associated with recurrence. Molecular subtype (P = 0.584) and Ki67 (P = 0.857) were not statistically associated with recurrence. Treatments of breast cancer including breast surgery (P = 0.603), axillary surgery (P = 0.273), and adjuvant radiotherapy (P = 0.664) were not identified as factors of recurrence to be associated with recurrence-free survival (RFS). In a multivariable analysis which included variables with a p-value of < 0.05 on univariable analysis, the clinical T stage (P = 0.320) and clinical N stage (P = 0.073) were not statistically significant (**Table 2**).

**Table 2:** Univariable and multivariable Cox regression.

Characteristics	Hazard ratio	95% CI	P value	Hazard ratio	95% CI	P value
**Age at diagnosis (years)**			0.141			
≤ 35	1.19	024 - 5.90	0.836			
36 - 50	1					
> 50	2.57	0.96 - 6.85	0.060			
**Clinical T stage**			0.049			0.320
cT0/cTis/cT1	1			1		
cT2	0.66	0.14 - 3.02	0.594	0.83	0.18 - 3.84	0.807
cT3	1.2	0.24 - 5.95	0.826	1.19	0.24 - 5.91	0.830
cT4	5.64	0.79 - 40.11	0.084	4.14	0.54 - 31.8	0.172
**Clinical N stage**			0.010			0.073
cN0	1			1		
cN1	1.9	0.45 - 7.97	0.379	1.75	0.41 - 7.38	0.449
cN2	2.9	0.77 - 10.94	0.117	2.89	0.76 - 10.93	0.119
cN3	10.93	2.44 - 48.94	0.002	7.35	1.47 - 36.82	0.015
**Molecular subtype at diagnosis**			0.584			
HR+/HER2-	1					
HR+/HER2+	1.75	0.36 - 8.45	0.488			
HR-/HER2+	1.15	0.24 - 5.56	0.861			
HR-/HER2- (TNBC)	0.75	0.14 - 4.09	0.738			
Ki67 at diagnosis						
< 20%	1					
≥ 20%	1.14	0.27 - 4.93	0.857			
Breast surgery						
Mastectomy	1					
BCS	0.76	0.28 - 2.10	0.603			
Axillary surgery						
SLNB only	1					
ALND	1.66	0.67 - 4.13	0.273			

BCS, breast-conserving surgery; SLNB, sentinel lymph node biopsy; ALND, axillary lymph node dissection; HR, hormone receptor; HER2, human epithelial growth factor receptor 2; TNBC, triple-negative breast cancer; RT, radiotherapy.

### Prognosis of pCR patients with clinicopathologic factors

**Figure 2:**
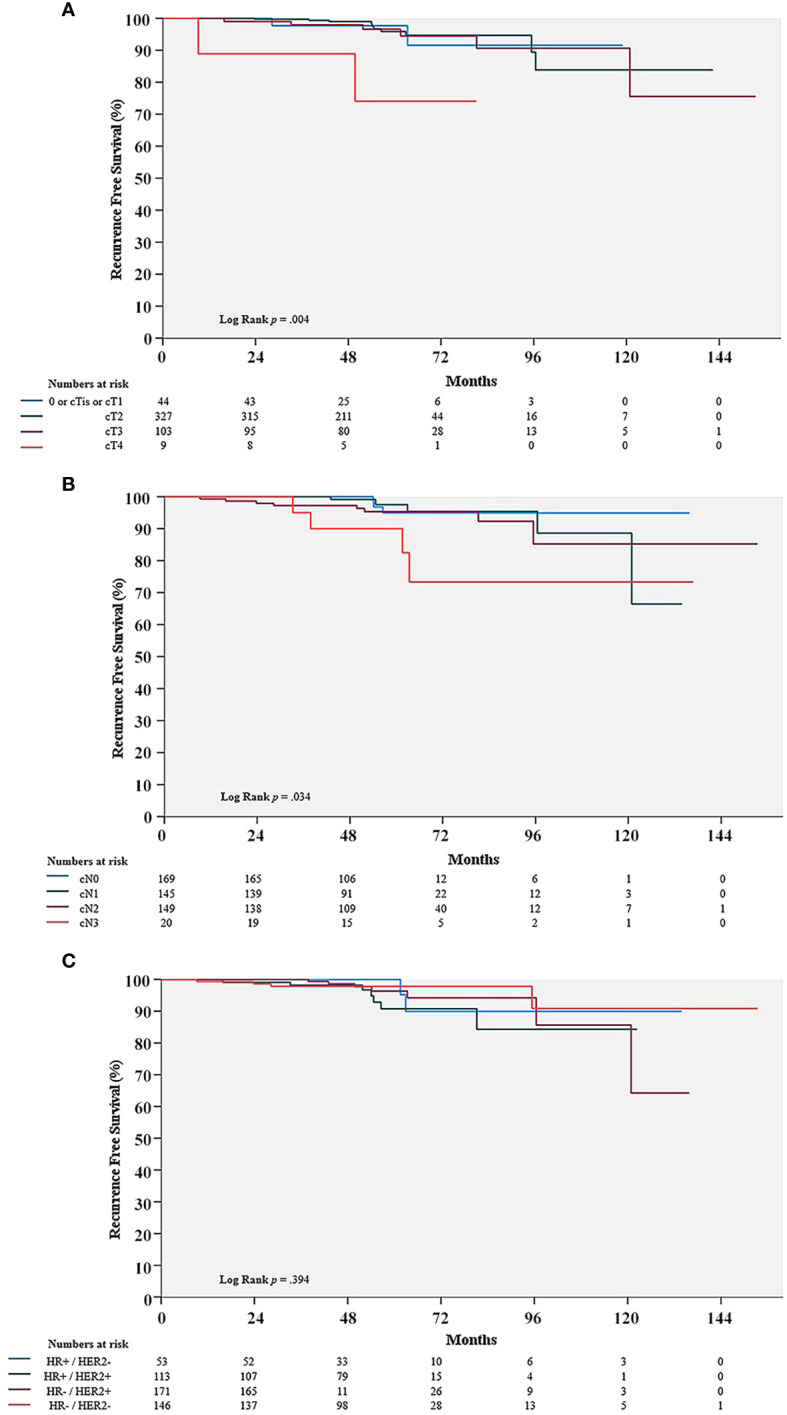
Comparison of recurrence-free survival (RFS) rate in the pCR group with cT stage **(A)**, cN stage **(B)**, and molecular subtypes **(C)**. pCR, pathologic complete response; HR, hormone receptors; HER2, human epithelial growth factor receptor 2.

The clinical T stage demonstrated a significant difference in the Kaplan-Meier curve for RFS (P = 0.004). The clinical N stage also exhibited a significant effect. (P = 0.034). However, molecular subtypes did not show any statistically significant effect on RFS (P = 0.394) (**Figure 2**).

### Clinicopathologic characteristics of patients with a recurrence and metastases

The mean follow-up of RFS for 480 patients achieving pCR was 26.5 months (range, 6.0-92.1 months). Out of 483 patients achieving pCR, 20 finally developed a recurrence of cancer. Out of these 20 patients, 2 (10%) had HR +/HER2 – tumors, 14 (70%) had HER2 + tumors, and 4 (20%) had TNBC. Only 2 (10%) had clinical T1 tumors and 3 (15%) had clinical N0 tumors without any overlap. The other patients (75%) had clinical T2-4 or clinical N1-3 tumors. Ten patients (50%) developed LRR, 14 (70%) underwent distant metastases and among them, 4 (20%) had both. Six patients (30%) in the recurrence group finally expired due to breast cancer (**Table 3**).

**Table 3:** Characteristics of patients with recurrence.

						State at recurrence			Initial treatment information			Expire
No.	Age	molecular subtype	cT	cN	Ki67	LRR	Distant metastasis	RFS (months)	Breast OP	Axilla OP	Adjuvant RT	
1	32	HR+/HER2-	cT2	cN1	≥ 20%		sternum, lung	16.6	mastectomy	SLNB only	–	–
2	50	HR+/HER2-	cT3	cN3	< 20%		sternum, lung, Rt paratracheal LN	37.8	mastectomy	ALND	+	+
3	52	HR+/HER2+	cT3	cN2	≥ 20%		brain	13.3	mastectomy	SLNB only	+	–
4	55	HR+/HER2+	cT3	cN2	≥ 20%	ipsilateral SCN	C6-spine, vertebral cervical LN	47.5	BCS	ALND	+	–
5	45	HR+/HER2+	cT3	cN2	≥ 20%		brain	6.3	BCS	SLNB only	+	–
6	56	HR+/HER2+	cT2	cN0	≥ 20%	ipsilateral breast	contralateral ALN	46.8	BCS	SLNB only	+	+
7	50	HR+/HER2+	cT2	cN1	≥ 20%	ipsilateral breast		8.9	BCS	SLNB only	+	–
8	58	HR+/HER2+	cT2	cN0	< 20%	ipsilateral SCN		45.0	BCS	ALND	+	–
9	56	HR+/HER2+	cT3	cN3	≥ 20%		brain	6.0	BCS	SLNB only	+	+
10	45	HR-/HER2+	cT2	cN1	≥ 20%		brain	20.5	BCS	ALND	+	–
11	38	HR-/HER2+	cT2	cN1	≥ 20%	ipsilateral breast		12.3	BCS	SLNB only	+	–
12	53	HR-/HER2+	cT4	cN2	≥ 20%	ipsilateral breast	contralateral ALN	30.8	BCS	ALND	+	–
13	44	HR-/HER2+	cT3	cN1	≥ 20%	ipsilateral breast		9.1	mastectomy	ALND	+	–
14	60	HR-/HER2+	cT2	cN3	≥ 20%		lung, mediastinum	20.5	BCS	SLNB only	+	–
15	52	HR-/HER2+	cT1	cN3	≥ 20%		brain	29.1	BCS	ALND	+	+
16	61	HR-/HER2+	cT2	cN0	≥ 20%	ipsilateral breast		49.2	BCS	SLNB only	+	–
17	55	HR-/HER2- (TNBC)	cT4	cN2	≥ 20%		brain	8.2	mastectomy	ALND	–	–
18	32	HR-/HER2- (TNBC)	cT2	cN2	≥ 20%	ipsilateral breast		92.1	BCS	SLNB only	+	+
19	42	HR-/HER2- (TNBC)	cT1	cN2	≥ 20%	ipsilateral breast/ALN	brain, paratracheal LN	12.8	BCS	SLNB only	+	–
20	60	HR-/HER2- (TNBC)	cT2	cN2	≥ 20%		brain	17.0	BCS	SLNB only	+	+

HR, hormone receptor; HER2, human epithelial growth factor receptor 2; LRR, locoregional recurrence; RFS, recurrence-free survival; OP, operation; RT, radiotherapy; SLNB, sentinel lymph node; Rt, right; ALND, axillary lymph node dissection; LN, lymph node; SCN, supraclavicular node; ALN, axillary lymph node; BCS, breast-conserving surgery.

## Discussion

In this retrospective study, we demonstrated that the clinical T stage and clinical N stage were relative to the risk of breast cancer recurrence after achieving pCR following NAC in univariable Cox regression analysis. In addition, the Kaplan-Meier curve demonstrated the association between these factors and recurrence-free survival after achieving pCR. However, these factors were not correlative with a risk of tumor recurrence after achieving pCR in multivariable Cox regression analysis. In previous studies, the predictive significance of pre-NAC clinicopathological variables in pCR patients has been primarily evaluated (20–22). These studies have demonstrated a relationship between the higher pre-NAC clinical stage and a higher risk of tumor recurrence after achieving pCR. Additionally, our results showed that pCR patients who had more advanced tumors in terms of pre-NAC clinical T or clinical N stages had worse outcomes although it was not statistically significant. Moreover, there is no significant difference of factors related in recurrence in patients with HER2-low (**Table S1**).

According to previously reported meta-analysis, pCR rates were varied by molecular subtypes, while the association between pCR and long-term outcome was controversial (7, 23). CTNeoBC (Collaborative Trials in Neoadjuvant Breast Cancer) pooled analysis showed that the frequency of pCR in patients with HR +/HER2 – tumor was the lowest, while the more aggressive subtypes, HER2 + tumors and TNBC, had increased frequencies of pCR; pCR rate of HR +/HER2 – tumor; up to 16.2% (95% CI, 6.3 – 19.3), HER2 + tumor; up to 50.3% (95% CI 45.0 – 55.5), and TNBC; up to 33.6% (95% CI, 30.9 – 36.4). Furthermore, it was showed that, although pCR was positively correlated with oncologic outcomes, it varied by molecular subtype. Compared to TNBC and HER2 + tumors, the prognosis of HR +/HER2 - cancers was marginally improved; hazard ratio for RFS and OS in HR +/HER2 – (0.49 (95% CI 0.33 – 0.71) and 0.43 (95% CI 0.23–0.71)) versus hazard ratio for RFS and OS in HR -/HER2 + (0.15 (95% CI 0.09 – 0.27) and 0.08 (95% CI 0.09 – 0.22)), hazard ratio for RFS in TNBC (0.24 (95% CI 0.18 – 0.33)). Whereas, some RCTs such as I-SPY2 study showed that hazard ratios for oncologic outcomes following pCR were consistent across all subtypes of breast cancer.; hazard ratio of RFS in HR +/HER2 – tumors, 0.14 (95% CI, 0.03 - 0.55); hazard ratio of RFS in HR +/HER2 + tumors, 0.18 (95% CI, 0.05 - 0.41) (24). Proportions of pCR achieving tumor in our study were different with molecular subtypes; 11.0% (HR +/HER2 –), 58.8% (HER2 +), and 30.2% (TNBC). RFS with molecular subtypes was not different and it was showed in **Figure 2C** (P = 0.394), which was similar to result of CTNeoBC analysis. Therefore, the association between achieving pCR and oncologic outcomes might still be debatable, necessitating additional studies.

In the literature, HER2 + breast cancer has demonstrated a higher risk of LRR and distant metastases even after achieving pCR (21, 25–27). Tanioka et al. showed that 88 (19.6%) of 449 patients achieved pCR after NAC and among 88 patients, 43 (48.9%) patients had HER2 + tumors. Through multivariate analysis, the HER2 + tumor was identified as a significant risk factor for recurrence after achieving pCR (hazard ratio, 5.0; P < 0.019) (26). In addition, Liedtke et al. showed that 255 (20.1%) of 1,118 patients who had TNBC received NAC and they had significantly higher pCR achieving rates, compared with non-TNBC patients (22% vs. 11%; P = 0.034) (28). In this study, the tendency of LRR and distant metastasis after achieving pCR was similar to previous studies; pCR 214 achieving rates in HER2 + tumor and TNBC were high. However, these tumors still were associated with a recurrence including LRR and distant metastases after achieving pCR. Among 20 patients who developed recurrence after pCR, 10 patients had LRR. Among them, most of them had HER2 + tumors (8 patients) or TNBC (2 patients), and there were no patients with HR +/HER2 – tumors. Among 14 patients who had distant metastases after achieving pCR, 9 patients had HER2 + type, 3 patients had TNBC, and 2 patients had HR+/HER2 – type. Furthermore, a pattern of expired patients due to breast cancer (6 of 483) was similar to that of the pattern of tumor recurrence after achieving pCR; HER2 + tumors (3 patients), TNBC (2 patients), and HR +/HER2 – (only 1 patient). This study supported the idea that HER2 + tumors and TNBC tend to achieve pCR and remain in a high-risk group, which has been constantly suggested in the literature (29, 30).

In previous studies, other risk factors except for molecular subtypes of tumors have been reported. Young age, clinical T stage, and clinical N stage were referred to as risk factors for recurrence including LRR and distant metastases after achieving pCR (31–36). According to Ishitobi et al., patients who were younger than 40 years at the time of diagnosis had significantly worse IBTR-free survival than those who were 40 years or older (5-year IBTR-free survival, 87.7 vs 96.9%; *p* = .002) (31). Li-Yun Xie et al. identified that among 1,913 patients who received NAC, 420 achieved pCR (22.0%), and clinical T stage and clinical N stage were associated with tumor recurrence in the pCR achieving patients after NAC (hazard ratio: 2.57, 95% confidence interval: 1.01-6.51, P = 0.047 for clinical T stage, and hazard ratio: 3.48, 95% confidence interval: 1.37-8.83, P = 0.009 for clinical N stage) (36). Comparatively, this study showed that age, clinical T, clinical N stage, and molecular subtypes were not statistically significant predictors of recurrence in patients who achieved pCR after NAC. A possible explanation for the different recurrent risk factors between this study and previous studies is the difference in the proportion of patients who received anti-HER2 targeted therapy. In this study, a portion of HER2 + patients did not receive anti-HER2 treatment because they were diagnosed before national insurance coverage of anti-HER2 targeted therapy. Thus they did not benefit from anti-HER2 treatment. Another explanation for age, clinical T stage, and clinical N stage are that the distribution of patients was unequal in the study groups. In the recurrence group, the total number of patients was only 20, which was too small to obtain a statistical significance, compared to the no recurrence group (n = 463). In addition, patients with severely advanced breast cancer such as internal mammary lymph node (IMN) metastasis, supraclavicular lymph node (SCN) metastasis, and inflammatory breast cancer were excluded in our study. On the other hand, in most of references cited, patients with these severely advanced tumors were included (26, 28, 31). It was suggested that including cases of severely advanced breast cancer in the study might make the difference between our study and references. Attentively, it was proposed that achieving pCR would be more important factor than other factors such as cT stage, cN stage, age, and molecular subtypes. In other words, the predictive factors of achieving pCR would be another factor of recurrence following pCR. According to the literature, the use of breast radiologic complete response (rCR) was proposed as another strategy to anticipate achieving pCR (37, 38). Woo et al. showed that breast rCR was a significant factor for a favorable oncologic outcome in previous studies. Among 1017 patients, 287 (28.2%) achieved breast pCR, 165 (16.2%) achieved breast rCR, 529 (52.0%) had axillary pCR, and 274 (26.9%) achieved axillary rCR. A breast rCR and pCR correlation showed a Cohen’s Kappa value of 0.459, and an axillary rCR and pCR correlation indicated a value of 0.384. However, due to rCR did not completely predict pCR, minimal residual tumor should be considered. Thus, factors of recurrence in the area of minimal residual disease should be discovered and further investigated.

Recently, progressive adjuvant treatments have been evaluated by RCTs such as the ExteNET trial. Adding neratinib, an irreversible pan-HER tyrosine kinase inhibitor, after neoadjuvant and adjuvant anti-HER2 treatment for patients with stage 2-3 HER2 + tumor resulted in favorable prognosis; hazard ratio: 0.58, 95% confidence interval: 0.41-0.82, P = 0.002 for 5-year invasive DFS, hazard ratio: 0.79, 95% confidence interval: 0.55-1.13, P = 0.203 for OS; but in HR +/HER + group the results were; hazard ratio: 0.60, 95% confidence interval: 0.33-1.07, P = 0.086 for 5-year invasive DFS, hazard ratio: 0.47, 95% confidence interval: 0.23-0.92, P = 0.031 for OS (39). However, despite achieving pCR is undoubtedly a significant purpose for patients who received NAC, the toxicity of these treatments should be not ignored (40). Thus, strict screening is mandatory to identify whether patients correspond to a high-risk group to minimize unnecessary adverse effects. Consequently, tailoring NAC for breast cancer patients is important to not only prevent recurrence but also avoid over-treatment for low-risk patients (41, 42).

This study had several limitations. First, as this study was retrospective, some parts of the data were missing and none were replaceable. Second, the study lacked a sufficient number of patients who had a recurrence and distant metastasis after achieving pCR. A single-center study design was another limitation of this study. Consequently, further study with a sufficient number of patients in a multicenter is expected to demonstrate a correlation with recurrence rate and insignificant factors such as age, clinical T stage, clinical N stage, and molecular subtypes. Nevertheless, some tentative conclusions were achieved from this retrospective study. First, this study attentively suggested that achieving pCR would be more important factor than other factors such as cT stage, cN stage, age, and molecular subtypes. Second, even though HER2 + tumors and TNBC have a high potential of achieving pCR, it seemed that these subtype tumors also had a high potential of recurrence. Thus, the escalated treatment might be required for HER2 + tumors and TNBC. However, it would also be important to tailor the treatment for each patient in order to prevent treatment toxicity. In conclusion, patients with HER2 + tumors or TNBC should be given additional treatments with a strict patient selection strategy to prevent over-treatment as well as achieve pCR.

## Data availability statement

The datasets presented in this study can be found in online repositories. The names of the repository/repositories and accession number(s) can be found in the article/**Supplementary Material**.

## Ethics statement

The studies involving humans were approved by Institutional Review Board (IRB) of Samsung Medical Center (IRB number: 2022-08-139). The studies were conducted in accordance with the local legislation and institutional requirements. Written informed consent for participation was not required from the participants or the participants’ legal guardians/next of kin in accordance with the national legislation and institutional requirements. Written informed consent was not obtained from the individual(s) for the publication of any potentially identifiable images or data included in this article because This study was approved without informed consent by IRB in SMC because this study was a retrospective study. It was stated in IRB approval (number 2022-08-139).

## Author contributions

Conceptualization: JR. Data curation: JC, DW. Formal analysis: JC, BC, JY. Investigation: SJ, DS. Methodology: HJL, YK. Software: JC. Validation: JR. Visualization: DW. Writing - original draft: JC, DW. Writing - review & editing: JC, HWL, JL, SK, SN, JR. All authors contributed to the article and approved the submitted version.
