# Supplementary material for: Risk factors of breast cancer recurrence in pathologic complete response achieved by patients following neoadjuvant chemotherapy: a single-center retrospective study
Source: Front Oncol. 2023 Oct 2;13:1230310. doi: 10.3389/fonc.2023.1230310 (PMC10577442; doi:10.3389/fonc.2023.1230310)
Supplement: Supplementary file 1 [file Table_1.docx]

**Table S-1** Clinicopathologic characteristics of patients with HER2-low.

| **Characteristics** | | **Recurrence No. (%)** | **No Recurrence No. (%)** | |  | **P value** |
| --- | --- | --- | --- | --- | --- | --- |
|  |  | **N = 2 (2.7)** | **N = 71 (97.3)** | **%** | |  |
| **Mean F/U duration (month)** | | 58.1 (23.8 - 136.7) | |  | |  |
| **Age at diagnosis** | |  |  |  | | 0.153 |
|  | ≤ 35 | 0 (0.0) | 13 (18.3) | 17.8 | |  |
|  | 36 - 50 | 0 (0.0) | 34 (47.9) | 46.6 | |  |
|  | > 50 | 2 (100) | 24 (33.8) | 35.6 | |  |
| **Clinical T stage** | |  |  |  | | 0.515 |
|  | cT0 or Tis or T1 | 0 (0.0) | 8 (11.3) | 11.0 | |  |
|  | cT2 | 1 (50.0) | 50 (70.4) | 69.9 | |  |
|  | cT3 | 1 (50.0) | 11 (15.5) | 16.4 | |  |
|  | cT4 | 0 (0.0) | 2 (2.8) | 2.7 | |  |
| **Clinical N stage** | |  |  |  | | 0.107 |
|  | cN0 | 0 (0.0) | 25 (35.2) | 34.2 | |  |
|  | cN1 | 1 (50.0) | 43 (60.6) | 60.3 | |  |
|  | cN2 | 0 (0.0) | 0 (0.0) | 0.0 | |  |
|  | cN3 | 1 (50.0) | 3 (4.2) | 5.5 | |  |
| **FNA of metastatic lymph node** | |  | |  | | 0.726 |
|  | negative by proven Bx | 0 (0.0) | 17 (23.9) | 23.3 | |  |
|  | positive by proven Bx | 2 (100.0) | 34 (47.9) | 49.3 | |  |
|  | Did not Bx | 0 (0.0) | 20 (28.2) | 27.4 | |  |
| **Hormone receptor** | |  |  |  | | 1.000 |
|  | positive | 1 (50.0) | 28 (39.4) |  | |  |
|  | negative | 1 (50.0) | 43 (60.6) |  | |  |
| **Ki67 at diagnosis** | |  |  |  | | 0.280 |
|  | < 20% | 1 (50.0) | 8 (11.3) | 12.3 | |  |
|  | ≥ 20% | 1 (50.0) | 61 (85.9) | 84.9 | |  |
|  | Unknown | 0 (0.0) | 2 (2.8) | 2.7 | |  |
| **Breast surgery** | |  |  |  | | 0.349 |
|  | Mastectomy | 1 (50.0) | 13 (18.3) | 19.2 | |  |
|  | BCS | 1 (50.0) | 58 (81.7) | 80.8 | |  |
| **Axillary surgery** | |  |  |  | | 0.455 |
|  | SLNB only | 1 (50.0) | 53 (74.6) | 74.0 | |  |
|  | ALND | 1 (50.0) | 18 (25.4) | 26.0 | |  |
| **Adjuvant RT** | |  |  |  | | 1.000 |
|  | Yes | 2 (100.0) | 68 (95.8) | 95.9 | |  |
|  | No | 0 (0.0) | 3 (4.2) | 4.1 | |  |
| **NAC regimen** | |  |  |  | | 1.000 |
|  | AC | 0 (0.0) | 4 (5.6) | 5.5 | |  |
|  | T | 0 (0.0) | 1 (1.4) | 1.4 | |  |
|  | AC+T | 2 (100.0) | 57 (80.3) | 80.8 | |  |
|  | ACTH | 0 (0.0) | 0 (0.0) | 0.0 | |  |
|  | TCHP | 0 (0.0) | 0 (0.0) | 0.0 | |  |
|  | others | 0 (0.0) | 9 (12.7) | 12.3 | |  |

F/U, follow-up; FNA, fine needle aspiration; Bx, biopsy; HR, hormone receptors; HER2, human epithelial growth factor receptor 2; TNBC, triple-negative breast cancer; BCS, breast-conserving surgery; SLNB, sentinel lymph node biopsy; ALND, axillary lymph node dissection; RT, radiotherapy; NAC, neoadjuvant chemotherapy; AC, Adriamycin Cyclophosphamide; T, Taxane; AC+T, Adriamycin Cyclophosphamide + Taxane; ACTH, Adriamycin Cyclophosphamide Taxane Herceptin(trastuzumab); TCHP, Taxane Carboplatin Herceptin(trastuzumab) Perjeta(pertuzumab).
